# Music as Environment: An Ecological and Biosemiotic Approach

**DOI:** 10.3390/bs5010001

**Published:** 2014-12-23

**Authors:** Mark Reybrouck

**Affiliations:** Faculty of Arts, Section of Musicology, KU Leuven—University of Leuven, Blijde Inkomststraat 21, PO BOX 3313, Leuven 3000, Belgium; E-Mail: Mark.Reybrouck@arts.kuleuven.be; Tel.: +32-16-32-48-86

**Keywords:** music as environment, sonic universe, Humboldt system, internal semantics, fetal auditory development, ecology, affordance, biosemiotics

## Abstract

This paper provides an attempt to conceive of music in terms of a sounding environment. Starting from a definition of music as a collection of vibrational events, it introduces the distinction between discrete-symbolic representations as against analog-continuous representations of the sounds. The former makes it possible to conceive of music in terms of a Humboldt system, the latter in terms of an experiential approach. Both approaches, further, are not opposed to each other, but are complementary to some extent. There is, however, a distinction to be drawn between the bottom-up approach to auditory processing of environmental sounds and music, which is continuous and proceeding in real time, as against the top-down approach, which is proceeding at a level of mental representation by applying discrete symbolic labels to vibrational events. The distinction is discussed against the background of phylogenetic and ontogenetic claims, with a major focus on the innate auditory capabilities of the fetus and neonate and the gradual evolution from mere sensory perception of sound to sense-making and musical meaning. The latter, finally, is elaborated on the basis of the operational concepts of affordance and functional tone, thus bringing together some older contributions from ecology and biosemiotics.

## 1. Introduction

Can we conceive of music as a sounding environment? And how does a listener cope with this environment? Can we rely on existing definitions of music and dealing with music, or is there need of broadening of the conceptual framework to describe the listening behavior that is common across human listeners? This contribution tries to give an answer to these questions by introducing an ethological framework that describes the listening process from a biological point of view, relying heavily on the concept of coping with the sounds. It stresses the need of clear definitions of general concepts, such as environment, sounding environment and sonic universe and provides an operational framework for the description of the process of coping with the sounds by relying on the older insights of ecology and biosemiotics.

The term sonic or sounding environment, first, has many definitions, depending upon the field of study. It can range from the environment of the embryo, the intimate environment of the infant, to the home, the concert hall, the city or expanses of wild nature, but researchers in each of these fields are all interested in sound. In an attempt to standardize these meanings, attempts have been made to provide definitions and criteria for classifying them [1,2,3] revolving basically around the concept of *soundscape,* which suggests exploring all the sounds in the environment in its complexity, ambivalence, meaning and context. The soundscape concept, as introduced by Schafer [4], was originally thought as an evaluation of noise and its effects on the quality of life but has evolved later to incorporate both the conditions and purposes of its production and reception. Having been the subject of much research and applications [3,5,6] it has furthered a kind of paradigm shift in the evaluation of environmental sound from “measurement by instruments” to “measurement by human persons” [1]. The concept of soundscape, in fact, explicitly includes a subjective component, namely the way in which the environment is perceived and understood by an individual or by a community [7] with a corresponding shift towards a more cognitive approach to environmental sounds as meaningful events that affect people [8,9,10]. This is exemplified in the considerations of a Working Group (ISO/TC 43.SC1) that was established to begin consideration of a standardized method for assessment of soundscape quality outdoors, incorporating not only the presence or absence of annoying sounds, but also the positive aspects of sound environments as perceived by people as well. There is, in fact, a wealth of different terms that are used to describe the soundscape, such as the acoustic environment, the sonic environment, the sound environment, the environment of sound, aural space, the natural acoustic environment and environmental sounds, sound ambient environments, ambient conditions, quiet areas, areas of high acoustic quality, city soundscape, the total ambient acoustic environment, the total soundscape, the acoustic soundscape and many others. A completely adequate and appropriate term that was suggested for the entity on which soundscape studies focus was the “acoustic environment as perceived or experienced and/or understood by people in context” [11].

Much early research has been directed to the study of noise and noisy environments with a special focus on city noise [6,12,13], but also here there has been a shift from the study of mere noisy spaces to a conception of the soundscape as resource, addressing not only noise avoidance, but rather sound quality of urban environments. Soundscape analysis, in this view, places sound in context with the listeners’ sensational reality depending on their socio-cultural background and the psychological dimension of the acoustical setting and with as major question “What does this feature of the environment mean to me?” [2,14]. It calls forth an *affordance-driven approach* to the study of sonic environment (see below), which means that soundscape analysis should combine the physical measurement of sound with a scientific investigation and evaluation of the community and individual perception of sound in terms of what it affords to the listener, thus relating human hearing and objective measurement. Acoustic environments, therefore, cannot be measured merely in terms of acoustic parameters, such as energy equivalent sound pressure levels (L_eq_) in A-weighted decibels (dB(A)).

What is needed, on the contrary, is a shift to more qualitative evaluations of sound quality with analysis techniques, which aim at deriving relevant inferences about how people process and conceptualize sensory experiences in an attempt to bridge the gap between individual sensory experiences and sociological representations of soundscapes [9].

Applying this to the realm of music calls forth a redefinition of its scope. Music, in fact, is a vibratory phenomenon that impinges upon the senses. The issue can be raised, therefore, whether we should adhere to a “humano-centric” web of culture that conceives music as a cultural artifact with little room for the natural [15] or as something that calls forth biological reactions to the sounds. The question brings together biology, humanities and music and is illustrative of the biology/culture and nature/nurture dichotomies, which have colored musical discussions for decades [16]. The biological position argues for wired-in mechanisms of reactivity to music as sound; the cultural position argues for responses that are the outcome of immersion in a culture. The antithesis, which was very strong at first, however, has begun to break down recently with developments in *evolutionary theory* and *ethological research*, which state that cultures may vary, but that the human capacity for culture appears to be a human universal [17,18]. Both culture and music are born out of man’s animal characteristics, which are rooted in the biology of perception and cognition—this is the *gene-culture coevolutionary theory* [19]—and this may be universal to a great extent [18,20,21,22]. It calls forth the issue of musical universals [23,24], which can be studied from the point of view of music-structural characteristics as well as from the dispositional machinery to deal with music. Arom’s definition of *anthropomusicology*, e.g., is typical of the first approach. In an attempt to provide a general description of music, he provides a list of (quasi-)universal human musical properties that are manifested in the whole body of known music of the world and which embrace at least four criteria: intentionality or an act of intentional construction; a formal process which detaches the music from the sound environment; a set of contrastive pitches, and, if the music involves more than one individual, modes of coordination between them [25]. It is possible, however, to broaden this list by relying not merely on music as a human-made phenomenon but also on more general mechanisms of coping with the sounding world.

## 2. The Acoustic Environment and the Sonic Universe

Music, in its broadest definition, is a subset of the *sonic universe*, which can be considered as the collection of sounding elements that represent the totality of sounds as a virtual infinity of possible combinations of individual vibrational events [26]. Sound, as a physical phenomenon, is caused by vibration, but not all vibrational events can be categorized as music and not all vibrations even make sound. We should distinguish, further, between distinct acoustic categories, such as noise and music, dependent upon the specific vibrational contents of the sounds. Periodic and complex vibrations, such as the sounds of musical instruments and the human voice, e.g., produce harmonics—the characteristic vibrational patterns that occur simultaneously with a fundamental tone and that are responsible for the richness of the sound—which are perceived mostly as pleasant and preferred auditory stimuli; noise, on the contrary, shows vibrations that result in irregular frequencies, with inconsistencies of tension, stress and configuration, and which mostly generate fatigue, stress, hyperalerting responses and startle reflexes in listeners [27,28]. This common distinction, however, can be questioned to some extent. Soundscape research, e.g., has provided many examples of sounds, which fit the definition of noise and yet were described as pleasant with the sound of flowing water as a typical example [29,30]. There is, further, also a subjective element that is related to the distinction between noise and music. The music of Stravinsky, Bartók, Stockhausen and others was perceived by some as noise, at least in the beginning, and Stravinsky’s “Le Sacre du Printemps” caused a near riot at the Paris Opera at its first performance. There are, in fact, significant personal, cultural and historical differences in this regard. It makes more sense, therefore, to start from another distinction, namely the difference between the use and meaning of the terms noise and sound in French and English [31]. The French word “bruit” (noise), e.g., was primarily used to refer to sources producing noise whereas “son” (sound) was used to describe acoustic phenomena as physical abstractions from the sources. They account for an important linguistic distinction to describe acoustic categories as related to different cognitive representations of the sounds [9].

Continuing this line of thought, the question can be raised, which elements are eligible for inclusion in the musical subuniverse. Should we conceive of natural or man-made sounds? The whole history of musical instrument building, e.g., has been one prolonged search for applying craftsmanship to raw materials in order to obtain musical sounds. Composers, however, are free also to incorporate non-musical sounds in their works, and to make something musical out of non-musical materials. As such, they can *musicalize* the sonic environment by giving semantic weight to sounding elements, which were otherwise neutral with respect to musical meaning. This is a process of *semiotization* of the sonic world, which is not limited to composers but which can be generalized to music listeners in general. Much depends, however, on the way these listeners deal with some of their environmental sounds. Is it enough to merely pick up musical sounds with a characteristic acoustic structure—suggesting that perception depends entirely upon information in the stimulus array [32,33]—or should we assume some cognitive processing and selective evaluation by the listener as well? The question is important as it cuts across the objective/subjective dichotomy. Musical sounds, in fact, point to both ways: they are part of an existing acoustic structure that is objectively there, but the pickup of sounds depends on acts of focal attention which are subjective to some extent.

### 2.1. The Concept of Environment

An environment, according to Lewontin, is all that surrounds. This broad definition by an evolutionary biologist simply means that there is no environment without an organism and no organism without an environment [34]. Environments of animals and men, moreover, are characterized by their most fundamental property, namely that they perceive and act in their environments. Perception and action, therefore, are not really separable at all [35,36] and most *subjective universes* or *environments* of animals and men are meaningful only to the extent that they are constituted within specific life activities. As such, they must be distinguished from the environment or the surroundings as these would appear to an indifferent observer without knowledge of the subjective meanings for a particular human or an animal. They constitute, so to say, phenomenal worlds or *Umwelts*, as von Uexküll lucidly has coined the term:

“The best time to set out on such an adventure is on a sunny day. The place, a flower-strewn meadow, humming with insects, fluttering with butterflies. Here we may glimpse the worlds of the lowly dwellers of the meadow. To do so, we must first blow, in fancy, a soap bubble around each creature to represent its own world, filled with the perceptions, which it alone knows. When we ourselves then step into one of the bubbles, the familiar meadow is transformed. Many of its colorful features disappear, others no longer belong together but appear in new relationships. A new world comes into being. Through the bubble we see the world of the burrowing worm, of the butterfly, or of the field mouse; the world as it appears to the animals themselves, not as it appears to us. This we may call the phenomenal world or the self-world of the animal.”

[37] (p. 5)

### 2.2. The Sonic Environment

Subjective universes cannot be described in merely objective terms. They imply a learning history and multiple interactions between an organism and its environment. This holds true, also, for the *sonic environment* of human listeners—considered as organisms—which is different at distinct phases of ontogenetic development, such as the intrauterine environment of the fetus, the extrauterine environment of the newborn and the natural and artificial sound environment of the developing child and adult listener. As such there is a whole history of sequential immersions in new environments: at first there is the heartbeat of the mother; other unfamiliar sounds are added very soon—Hicks has coined this the *uterine symphony* [38] (p. 31)—and from the 24th week on, the unborn child is immersed continuously in the very noisy environment of the pregnant abdomen and uterus; after birth, during the transition from intrauterine to extrauterine life, one of the most stressful changes is the loss of rhythm that the fetus has become accustomed to through months of being exposed to maternal movements, breathing, and heartbeat [39] and as neonates grow older, finally, they are embedded in increasingly challenging and complex sonic environments, which may be natural or artificial ones. The latter, especially, have become very pervasive in the last decades, with a sonic soundscape being shaped increasingly by traffic noise, industrial sounds and noises of work environment and even music. As such, there is a noticeable evolution, which was described by Schafer as a transition from a Hi-Fi to Lo-Fi Soundscape. To quote his words:

“A hi-fi system is one possessing a favourable signal to noise ratio. The hi-fi soundscape is one in which discrete sounds can be heard clearly because of the low ambient noise level. The country is generally more hi-fi than the city; night more than day; ancient times more than modern. In a hi-fi soundscape even the slightest disturbance can communicate interesting or vital information. The human ear is alert, like that of an animal. […] In a lo-fi soundscape individual acoustic signals are obscured in an overdense population of sounds. The pellucid sound—a footstep in the snow, a train whistle in the distance or a church bell across the valley—is masked by broad-band noise. Perspective is lost. On a downtown street corner there is no distance; there is only presence. Everything is close-miked. There is cross-talk on all the channels, and in order for the most ordinary sounds to be heard they have to be monstrously amplified. In the ultimate lo-fi soundscapes the signal to noise ratio is 1 to 1 and it is no longer possible to know what, if anything, is to be listened to.”

[40] (pp. 32–33)

The lo-fi soundscape, according to Schafer, is the common soundscape of today. His definition of the term, however, is problematical to some extent, as it reflects his own intuitions rather than empirical and scientific grounding. More recent soundscape research, in fact, has provided more fine-grained evidence of the perceptual structure of urban structure and industrial settings [6,9,41], but their noisy character is, of course, not easy to deny. But not all sonic environments of today are lo-fi soundscapes. There are quiet places and environmental biotopes in the countryside that may be defined unambiguously as hi-fi soundscapes. And also in the city, it is possible to locate hi-fi places, both at a public and individual scale. The concert hall, e.g., has made concentrated listening possible, and even the usage of earphones has provided a newly recovered “private space” that is commonly referred to as *head space*, to use a popular expression referring to the geography of mind [40] (p. 35). The influence of auditory social media, such as headphones, cellphones or iPhones, further, should be considered here as well. It is difficult at this moment to anticipate their effects on the perception of sonic environments as they create a new sonic environment at a micro scale that imposes on the larger sonic world of room, home, school, street and even the concert hall. They can even cut listeners off from the social and physical environment. Research on these effects, however, has just begun [42].

Sonic environments, in sum, are not univocal for all listeners, who can largely choose what to listen to and who can even modify their sonic world to some extent. They perceive only what is perceptually present, being there for them to be perceived, but involving also perceptual consciousness as a special style of access to the sonic world [43].

### 2.3. Music as Sounding Environment

Music, considered as a phenomenal subuniverse or sonic Umwelt, can be defined as a collection of subjective meanings imprinted upon a subset of the sonic world, including all the meaningful aspects of the sounding world for a particular listener. Taking this position as starting point means that the perceived qualities of the sounding world are not to be considered merely as objective characteristics of the sounds themselves, but as attributions that are acquired by the sounds having entered into diverse relationships with the listener. Or stated in ecological terms of *organism/environment interaction*: the listener—considered as an organism—fits the world to itself, ascribing functions to the objects it encounters and integrating them into a coherent system of its own [35]. The environment, in this view, is merely the projection or mapping out of the organism’s internal organization onto the outside world [34].

Music, further, has the possibility of being structured by the listener with the listening process being defined as a temporally extended, exploratory activity that brings together perception, manipulation, and appropriation of different sonic affordances offered up by sonic invariants that are present within the music. Krueger calls this the *world-making* power of the music as “a sonic world that affords possibilities for creating, organizing, and regulating listeners’ experiences, emotion regulation and social coordination”. Music, in this view, can be considered as a tool that is appropriate to construct different forms of self-experience and social relatedness [43] (p. 7).

This world-making power of musical environments has been studied extensively in neonate studies and in music therapeutic contexts, where sonically inviting aural spaces let the infant perceive music as something to be attended to and to cope with in an interactional way. Music, as a sounding environment, is acoustically unlike any other sound: it is more pleasant, soothing, and interesting than noise and uses highly preferred frequencies and harmonics selected through centuries of refinement and development of specific musical styles [44]. It can entrain, moreover, the infant to regulate its internal bodily states and expressive movements with positive recurrent features of the musical environment [44,45] [46] (p. 79). This holds true, of course, for some specific kinds of music if at least some sonic invariants are present, such as textural qualities and temporal regularities of sonic patterns, melodic as well as rhythmic ones [46] (p. 85). Most of the music that is used in the neonate context, therefore, is texturally soothing, constant (no abrupt modulations of volume, tempo, *etc.*) and relatively unchanging (exhibiting a temporally predictable melodic and rhythmic pattern) in order to reduce alerting responses by providing confidence [28,44]. Though such music may be sonically interesting by using a single singing voice, light rhythmic emphasis, constant rhythm and volume, and melodies in the higher vocal ranges, it is mostly perceptually undemanding so as to become an environment that the infant can perceptually conquer, as it were, by experientially understanding it and by perceptually gearing onto the predictable patterns in the music. As such, it manifests a kind of predictability that affords comfort, as well as a kind of mastery over the music, thus procuring security and stability [45].

Music as environment, however, is not to be restricted to the safe environment of the neonate. As the child grows older, its sonic environment is getting more unpredictable and complex, as both nature and culture provide huge bodies of structured sounds that can be raised to the level of music. Much depends, however, on the way how children learn to make sense out of the sonic world, relying not only on highly evolved genetically based biological mechanisms but also on supra-instinctual survival strategies that have developed in society and have been transmitted by culture. The latter, as a rule, require consciousness, reasoned deliberation and willpower with response selections that are no longer reducible to the functioning of a set of neural circuits in the older structures of the brain.

## 3. From Sound to Music: Discretization and Semanticity

Music and noise are qualitatively distinct, albeit not in a radical way. Sounds can be either noisy or musical, dependent on several criteria of psychophysical processing, such as harmonic structure, consonance and dissonance calibration [47,48]. Consonance—more specifically tonal consonance—refers to the peculiar sensorial experience associated to isolated tone pairs with simple vibration frequency ratios; dissonance, on the contrary, refers to inharmonic or complex vibration frequency ratios [49,50,51]. Ordinary listeners, further, can easily distinguish consonant from dissonant pitch combinations, equating these notions with a feeling of pleasantness (consonance) or unpleasantness (dissonance) [49,52].

The actual mechanism underlying these valuations arises from the roughness created at the level of the basilar membrane in the inner ear. Due to overlap in vibration patterns, the resolution of pitches of different frequencies is compromised, leading to the phenomenon of beating and a corresponding perception of roughness. The criterion of consonance, however, does not explain exhaustively the distinction between musical and noisy sounds. There is also the criterion of regularity and periodicity with musical sounds resulting from regular, periodic vibrations and noise resulting from non-periodic statistically random vibrations [53]. Sounds, in this view, have been considered as noise if their originating frequency is non-periodic and therefore of no determinate pitch, thus lacking purity in articulation [54]. This may be helpful in distinguishing between musical sound and noise in acoustic terms with noise being considered as undifferentiated sound. The acoustic description, however, is not the whole story. There is now a whole *ontology of noise* [55], which goes beyond its description as an auditory phenomenon. According to Sangild [56] (pp. 12–13) there are at least three basic definitions of noise: a musical acoustics definition relying purely on physics, a communicative definition based on distortion or disturbance of a communicative signal, and a definition based in subjectivity. We can in fact say that what is noise to one person can be meaningful to another and that what was considered unpleasant sound yesterday is not considered this way today.

As such, there has been a shift in the valuation and use of noise in music as well [57,58]. Starting with Luigi Russolo’s “L’Arte dei Rumori” (The Art of Noises) [59], there has been a major development in exploring the possibilities of using noise as musical material. Russolo experimented with noise machines, other composers, such as Avraamov used the urban sonic environment to compose a “Symphony of Sirens” in 1922 that was performed using the sounds and movements of human crowds, machine guns, cannons, factory sirens, airplanes, hydroplanes, trains, battleships, and a steam whistle across the spaces of Baku in Azerbaija [60]. There is, in fact, a whole history of noise in music, from experimental music of the early 20th century, the experiments of John Cage and Erik Satie, the emergence through industrial music, punk, free jazz and bands like Throbbing Gristle and the Boredoms since the mid 1970s to the Japanese noise music and glitch electronica of today.

There is, further, a tension between an increased prevalence of anthropomorphic noise in our daily environments and what is commonly considered as natural sounds. In contrast to human-made noise that was mostly absent during much of evolution, natural sounds can be defined as environmental sounds that are not generated by human-made machines. They include sounds of footsteps, wind, fire and rain, animal vocalizations, including human speech and other sounds generated for communication by animals (e.g., stridulation in crickets, buttress drumming by chimpanzees) and instrumental music by humans [61]. Animal vocalizations, especially, are dominated mostly by sustained harmonic sounds, in contrast to environmental sounds, which are dominated mainly by transient sounds. Speech sounds, on the other hand, are characterized by rapid variations in spectral distribution and temporal pattern with a change in the harmonic spectrum with distinct maxima (formants) in voiced sounds to filtered noise in fricative sounds [62].

Most behaviorally important sounds—animal and human vocalizations in particular, but also musical sounds—have as prominent feature their harmonic spectra [63,64] with central neural networks of the listener being preferentially attuned to consonant intervals because of their prevalence or biological significance in the environment [65,66,67]. All sounds, in fact, which are characterized by aspects of harmonicity and regularity, can easily be set apart from the acoustic background as a whole. As such, they involve an aspect of *self-similarity* in the sense that they can be recognized as such—sounds as sounds—which makes them apt for processes of *differentiation*, *discrimination* and *identification* [68].

### 3.1. Music as a Humboldt System

Musical sounds—in the above definition as natural and differentiated sounds—mostly are harmonic sounds. They are easily detectable and have combinatorial possibilities, both in a simultaneous and successive way. Music, in this view, achieves pattern richness on the basis of combinations of small sets of discrete elements—discrete durations in the temporal domain and sets of discrete pitches in the frequency domain—which yield resulting patterns that are unique, identifiable and recombinable. As such, it is possible to conceive of music as a *Humboldt system*, *i.e.*, a system that makes infinite use of finite media [69] and which is based upon the combination of discrete elements, as illustrated by the *particulate principle* of self-diversifying systems [70]. This principle holds that structures, which have an infinite range of possibilities, are based on particles, which can be combined. Several independent natural systems, such as chemical interactions, biological inheritance and human language, appear to exhibit change by a process of variation and selection of dynamically stable, particulate units.

The setting apart of specific sounds from the acoustic background is a first step in the structuring of the environmental sounding flux. It calls forth a process of *discretization* of the sonic universe, as is obvious in the domains of language and music where phonemes and musical sounds can be used in a combinatorial way. It should be borne in mind, however, that originally all sounds are indissolubly tied to the mechanisms, which produced them so as to become uncounterfeitable and unique. When not recorded by means of technological devices, each sound occurs at one time and in one place only. Even when they may bear resemblances to one another, such as the phonemes of language or the pitched sounds of musical instruments, they are not identical in a strict sense [40] (p. 34).

There is, further, a distinction to be drawn between *combinatorial* and *creative emergence*, the former referring to the novelty that results from fresh combinations of pre-existing elements [69,71], and the latter to “*de novo*” creations of new kinds of elements [72]. The distinction, however, is gradual rather than qualitative. Creativity in music is combinatorial but it is creative only to the extent that the elements and their combinations yield a product that can be perceived as something new. Most music, moreover, just as language, starts from a set of pitches or pitch combinations, durations, tone colors, *etc.*, but it is possible to go beyond the constraints of a limited set of pre-existing elements. Music, in fact, can incorporate all kinds of sounds—artificial and natural—in its texture, and there are so many idiosyncrasies and subjectivities in both the production and reception of music that the mere claim of a limited set of basic elements in music seems to be problematic. Yet, there are some universal musical properties, which are reducible to *psychophysical commonalities* between disparate tonal systems, and to the major role of relatively unchanging biological processes of aural perception in discovering patterns of sound.

As such, it is possible to conceive of basic sonic constituents, which are recognizable as such. There is a distinction, however, between the acoustic level of these constituents and their meaning, as exemplified most typically by the distinction between the *phonetic* and *phonological* level of description in linguistics. Phonetics, as a part of the study of speech, is a discipline that investigates how speech sounds are produced and perceived. It includes the study of the vocal tract and its neuromuscular machinery as well as the hearing apparatus and its underlying neurological structures. It can be considered the hardware that implements the control signals that originate from the phonological component. Phonology, on the other hand, is related to the logical and functional structure and behavior of speech sounds. It concerns the stored contents of the lexicon and the knowledge about their mutual relationships rather than the operations needed for their reception and production. Both levels, however, involve each other, as the phonological units and processes are shaped to some extent by the physical and physiological structure of the speech mechanism [73]. This is obvious from the domain of *descriptive* or *taxonomic phonetics*, which consists of a set of more or less objective anatomical-physiological descriptors of speech sounds—such as neuromuscular, anatomical, aerodynamic, acoustic, and auditory principles of speech sound production—and which could be pursued for giving answers to some phonological questions.

### 3.2. Internal Semantics and the Computational Approach

The phonetic/phonological distinction holds true for the basic constituents of speech. There is, however, a major distinction between *music* and *language*. Being combinatorial systems with discrete elements that can be differentiated from each other, they both rely on basic constituents that are reducible to discrete slices of temporal unfolding—the phonemes and morphemes of language and the individual sounds of music—and combinations of them, which may function as basic building blocks. The building blocks of language can be reduced to a finite set of elements—the lexicon—and a number of rules for combining them in well-formed ways. This lexicon, together with the grammatical rules, constitutes a finite system, which is characterized by *external semantics*, which means that the elements refer to something outside of the written or spoken text. This is not the case, however, for music, which can be considered as relying on a self-referential or *internal semantics*. Musical sounds—and combinations of them—do not refer to something outside of the music. Music, therefore, is *self-reflective* in referring only to itself, thus emancipating the sounds from any external and denotative meaning.

This self-reflective way of thinking about music has found its strongest expression in the *computational* approach. Computations, as a rule, are considered mainly from a symbol-processing point of view with as basic idea formal symbol manipulation by axiomatic rules, with a complete conceptual separation between the symbols and their physical embodiment. In this formal conception symbols and rules must be free of all influence other than their internal syntax, which means that there is no need to specify any specific sets of observables or to verify any truth-values with respect to the external world. Music, however, is also a sounding art, and to have meaning, its elements must be informally *interpreted*, *measured*, *grounded* or *selected* from the outside by a listener who functions as an external observer. Listeners, however, have a lot of freedom in delimiting the elements to which they can focus attention. As such they can apply symbolic labels to any possible delimitations of temporal unfoldings, which can be denoted in an act of mental pointing, and conceive of them as discrete things with “unit character”. The temporal unfoldings, however, can have a continuous representation as well, to the extent that they reflect the temporal articulation of the sound.

The delimitation of elements, further, is not totally free, as there are ecological and psychological constraints, which narrow down the temporal window of the sounding elements that listeners may mentally point at. There is, e.g., a difference between high-frequency or high-resolution processing of the sound (in the range of about 10 milliseconds) and the processing in terms of perceptual units (in the range of 2–3 s), which allow event identification over time [74,75], allowing a transition from a phenomenological description of the sound in acoustic terms to event perception and discrete labeling of temporal events.

There is thus an element of *sense-making* or *semiotization*, that addresses two approaches: *symbolic thinking* and *sensory experience*. Both are related to the representational format, which is either discrete or continuous. Continuous or analog representation is time-consuming and proceeds in real time; discrete-symbolic representation is proceeding in a much more economic way, also called outside of time. It reduces temporal unfoldings to single representations with an all-or-none character, which lend themselves to symbolic computations, which can be carried out on them. Both approaches, further, are not opposed to each other, but are complementary, calling forth the possibility of a combined analog-discrete approach. It means that music can be represented in two ways, namely discrete (e.g., stave notation) and continuous (e.g., recorded audio), and that humans can process music either as symbolic thinking or as a sensory experience. The idea is not new, but using these dichotomies to shed light on each other is interesting. It challenges the common practice of much musicological research to study music as a structure that is conceived outside of time (e.g., the score) in favor of an approach that describes listening as a real time experience [68]. Dealing with music, then, is dependent upon the continuous sonorous unfolding which proceeds in linear time as well as on its symbolic and discrete counterparts, which can be handled in a kind of virtual simultaneity. Or stated in other terms: music can be considered a kind of distributed substrate with discontinuities and focal allocations of semantic weight [76].

Music, in this view, is a time-consuming and sounding processual event that impinges upon our sensory and perceptual apparatus with its primary meaning being experiential [77] in relying heavily on the process of *sentic modulation* [78] which is a general modulatory system that is involved in conveying and perceiving the intensity of emotive expression by means of three graded spectra: tempo modulation (slow-fast), amplitude modulation (soft-loud) and register selection (low pitch-high pitch), somewhat analogous to the well-known rules of prosody.

## 4. Dealing with Sound and Music: Phylogenetic and Ontogenetic Claims

Aural perception requires the processing of sound vibrations in ways that are both innate and acquired as the outcome of development over time. As such it calls forth *ontogenetic* claims, which are manifest most typically in the auditory development of the fetus and the neonate. The newborn, in this view, is not to be considered as a blank slate but as the product of a learning history, which started already in the womb. Besides this developmental history, however, there is also a lot of hard-wired reactive behavior, which is geared by some *phylogenetically* older functions of the brain. This is the case, e.g., for brain stem reflexes and subcortical processing (the level that is situated anatomically below the higher functions of the brain which are situated in the cortex of the brain) of the sounds. A typical example is the “startle reflex”, which is a defensive and fast emotional response to sudden or threatening stimuli, such as a sudden dissonant event. It is not just a brain stem reflex—as has long been thought—but a reflex-like reaction that can be qualified as a subcortical reflex [47]. Ample evidence from neuroimaging and lesion studies has shown that subcortical structures, such as the parahippocampal gyrus and the amygdala, are involved in emotional responses to dissonance [79,80,81,82].

It thus seems that there is a reflex-like response to dissonance, which may be innate, together with a preference for consonance, which is also not dependent on prenatal or early postnatal experience. Hearing newborns from deaf parents, e.g., prefer consonance to dissonance [65], which points into an innate mechanism without auditory learning history. It has been shown, moreover, that even musicians who consider dissonance to be highly pleasant, exhibit enhanced electrodermal activity in response to dissonant music as compared to the consonant versions of the same music [48]. The mechanism is probably linked with the primary function of the auditory system as an adaptive functionality for signal detection—to estimate the size of objects or to assess sound sources and their characteristics—and which may be helpful to survive in a threatening environment.

### 4.1. Fetal Auditory Development

Auditory capability is one of the earliest discriminative abilities of the fetus, which has been shown to react in an increasingly sophisticated way to sounding stimuli already in its intrauterine phase of development [83]. Its development shows consecutively an increased fetal heart rate response to loud sounds (18 weeks of gestation), initial inconsistent responses to sound (25 to 27 weeks), consistent responses to auditory stimuli (29 weeks), initial hearing of maternal sounds and responses to them, and a beginning of discrimination among speech sounds, particularly with regard to pitch and rhythms (30 to 35 weeks) [84]. The full-term infant, further, responds to auditory stimuli at birth with a consistent head turn, particularly to higher frequencies at a moderate decibel level [85]. Having processed much auditory information during prenatal development, the infant also recognizes the voice of its mother at birth, prefers the voices of women and recognizes stories and melodies that were heard during the final trimester of fetal growth, with a preference for its native language [86,87,88].

As such, the auditory system seems to rely on genetically pre-wired universal processing dispositions, which are shaped also to some extent also by the intrauterine learning history and the auditory exposure from birth, which is critical to the further development and specification of the auditory abilities [44]. There is, further, a dynamic tension between music-specific and general-acoustic processes of auditory discrimination, but it should be borne in mind that the auditory system was devised primarily as an instrument for the detection and localization of distant acoustical disturbances, and as a contributor to the identification of the disturbing events, mostly within an action-oriented behavior. Acute perception, therefore, seems to have an evolutionary advantage in providing better fitness for interactions with a given environment. The end result of this screening behavior, however, does not merely depend on the relationship between the physical properties of the stimulus and the efficiency of the activated auditory system, but is weighed by the listener as an organism through simultaneous evaluation of the stimulus as a releaser of purposive behavior [89] (p. 231).

There is thus an aspect of *signification* in the process of dealing with sounds, which goes beyond a mere dispositional machinery for signal detection and psychophysical processing of the sounds and which stresses the role of the learning history that starts in the womb and which is related to the neurologic development of the fetus and the newborn infant. In the third trimester of fetal development, e.g., the human fetus adds 250,000 neurons per minute in the developing brain with the most critical period for the growth of human nervous cells (dendrites of the cerebral cortex) occurring between 20 weeks gestations and two years of age. Each newborn infant, therefore, must self-construct his cortical growth from post birth experiences, thus creating its own unique neurological connections and manifesting a lot of neural plasticity as well [44].

### 4.2. Neonates and Music-Related Sounds: Motherese and Infant-Directed Speech

The neural plasticity, which is so typical for neonates, is reflected also in the way they cope with music. As a rule, they seem to respond to music in rather sophisticated ways, perceptually speaking, by exhibiting a selective orientation to musical sounds, along with a more fine-grained critical discrimination of particular musical sound features, both of which are enacted via sensorimotor (*i.e.*, vocal and gestural) engagement with the music [90]. Caregivers all over the world have capitalized on this disposition by providing special kinds of music (lullabies and playsongs) and even special kinds of speech—called *motherese*, or *parentese—*with song-like qualities that seem to be very similar across all cultures. [91,92]. They talk to preverbal infants in a special way [93,94] and infants prefer to listen to this infant-directed speech to adult-directed speech [95,96,97]. This “baby talk”, which seems to be important for the cognitive development of the child by influencing cortical connections in the brain as the result of positive experience [98] seems to provide the right kind of environment during the first years of life. It tends to be higher in pitch, more rhythmic, and, most important for mediating infant preferences; it contains slower, more exaggerated pitch contours than adult-directed speech. Its common characteristics include extended vowels, mellifluous sound, narrow pitch range that rises for stimulation and falls for pacification, and repeated pitch contours [44,92,94,96,99,100,101,102]. In a sense, infant-directed speech could thus be called “musical speech”, because it differs from adult-directed speech in its prosodic or musical characteristics.

Both infant-directed music and song-like speech, further, seem to be classified among the primary and preferred stimuli of the early sonic universe. They provide intentional environmental sounds, which can be distinguished from the ambient noise, and which have shown to have positive physiological and behavioral effects on premature infants and neonates. There are, moreover, also simple psychoacoustic considerations, which might explain the preference of infants for some of the acoustic parameters that specify this specific sonic universe. High-pitched voices, e.g., sound less rough because they have fewer harmonics that interact within critical bands on the basilar membrane of the inner ear [103].

Infants who possess prelinguistic perceptual skills are thus highly sensitive to music-related speech sounds and music by tuning-in to subtle shifts in vocal timbre, tempo, and volume variations. They are also able to negotiate and share a mutual communication with their caregivers by constantly adapting their tempo, intensity, motion, shape, and contour of their sounds, movements, and gestures [104]. The newborn’s earliest interactions with their caregivers, for short, exhibit a distinctively musical character, which extends also to the expressive sound features of music. Young infants, in fact, are able to pick out not only holistic musical patterns but also fine-grained, formal properties of music, such as pitch, melody, tempo, and musical phrase structure, which all can be helpful in carrying expressivity and emotional content [105].

## 5. From Sound to Meaning: Ecological and Biosemiotic Claims

Dealing with sounds involves a process of sense-making that involves phylogenetic and ontogenetic claims. The latter, however, are dependent upon a learning history, which is highly idiosyncratic, as individual listener must face different sonic environments at different moments in their life. Yet, even this particular behavior exhibits some genetically-determined characteristics, which bring some benefit to them and which can be explained best in *ethological terms*. Ethology, which can be defined as the biological study of stereotypical behavior, is characterized by description of observable phenomena, with the aim to reduce the enormous variety of animal behavioral repertoires to simple explanatory mechanisms, which have been coined by Huxley the major problems of biology, namely *causation*, *survival* value and *evolution*, and to which Tinbergen has added also the problem of *ontogeny* [106].

Most of the ethological explanations of today are expressed in evolutionary terms, focusing on behaviors that are exhibited in quite analogous ways in the same or similar circumstances [107]. Being species-specific or common to a variety of species, they mainly contribute to the survival or fitness of the species and revolve around how animals cope with their environment. As such, they should be described in observational terms in order to answer the vague and general ethological question: why do animals—and thus also listeners, as human animals—behave the way they do? There is need, therefore, of a preliminary descriptive stage that returns to observation and description in order to analyze or interpret the overwhelming variety of puzzling behavior patterns. Ethology thus has provided data, methods, and theories that can help in understanding how humans cope with their environment. It is a research program that is pretty much analogous to the contributions from *ecology* and *biosemiotics*. The translation to the realm of music, however, mostly still has to be done [77,108,109].

### 5.1. Organism and Environment: The Ecological Approach

Starting from Haeckel’s definition of ecology as the science of the relations between an organism and its environmental outer word, and Kull’s related term of *ecosemiotics*—as the study of the semiotic interrelations between organisms and their environment [110,111,112]—it is possible to conceive of the process of dealing with music in *ecological* terms as “coping with the sounds”. This means that the way that listeners make sense of music is determined both by the characteristics of the listener as an organism and the music as environment. There is, however, not yet a major tradition of thinking of music in ecological terms [77,109,113,114] as most studies in ecological perception have been concerned with visual rather than with auditory stimuli.

The ecological program was mapped out by Gibson [32,33] who claimed that perceivers search out actively information, which becomes obtained information by leaning on *perceptual systems* rather than on *senses*. Senses, in this view, do not simply function to arouse sensations but pick up information, which is already structured and ordered as part of an organism-environment ecosystem [32] (p. 47). As such they should be conceived as perceptual systems, which are tuned to the information that is considered to be useful, stressing the reciprocity of the organism and its environment, hence, the role of key concepts, such as *attunement*, *reciprocity* and *resonance*, and the corresponding perceptual processes of detection, discrimination, recognition and identification. They all stress the role of *direct perception* [32,33,115], which claims that perception is a form of noninferential awareness without the mind intervening actively in the process. It involves direct contact with the sensory stimuli with reactions that are elicited in a lock-and-key approach, by applying conceptual knowledge that has been assimilated in the cognitive structure of the listener as the outcome of previous interactions with the sounds. As such, it calls forth the *principles of reality* and of *cognitive economy*, which means that there really is something “out there”—this is the “empiricist” or “realist” claim of perception—ready to be picked up an to be processed in a much more economical way. It has the advantage of speed of processing, which, in turn, has adaptive values for surviving in case of threatening situations.

The same holds true for dealing with music. Making sense out of the perceptual flux is not reducible to a kind of naive realism with acoustical or auditory listening as the only processing mechanism [116]. What matters is not merely an acoustical description of the continuous flow of matter in the physical world, but the way how human listeners structure this flow, and this is the basic tension between the “bottom-up” and “top-down” approach with the mind applying discrete labels (top-down) to a continuous unfolding (bottom-up). What listeners are listening to, therefore, are not sounding things, but “things as signs”, which shape their world.

### 5.2. Affordances and the Functional Approach

Conceiving of sounding stimuli as signs involves the introduction of the listener as an active searcher for meaning. It stresses the role of interaction between an organism and its environment, or in musical terms, the role of the sensorimotor engagement of the listener and its surrounding sonic universe.

The critical element in this approach is the sensitivity to functional characteristics of the environment. Animals and organisms, in general, perceive objects in their environment in terms of what they “afford” for the consummation of behavior, rather than in terms of their objective and perceptual qualities. The same claim was furthered by Gibson in his ecological psychology, as evidenced in the concept of *affordance*, which refers to environmental supports for an organism’s intentional activities: “*The affordances of the environment are what it offers the animal, what it provides or furnishes, either for good or ill*.” [33] (p. 127); [117].

Affordances, in this view, are subjective qualities that render them apt for specific activities, such as supporting locomotion, concealment, manipulation, nutrition and social interaction for the animal [33]. Numerous examples can be given, such as the surface of water as support for water striders or storks that nest on top of chimneys. As such, they are, first of all, environmental supports for goal-directed action, which are specified in relation to the activity-repertoire and skills that are unique to different animals. Animals’ sensitivities to different affordances, therefore, are both a function of their biology and their developmental and experiential history, which means that only an animal with a certain kind of anatomy and capacity for movement can see, e.g., a doorknob as graspable and pullable. Animals lacking this anatomy and motor capacity will not perceive these particular affordances [45].

Affordances, however, are not merely subjective. Being real and objective, they provide a conceptual tool that goes beyond the objective/subjective dichotomy by claiming that there is no outside standing over against an inside, but only ways to classify experiences [118]. As Gibson puts it:

“… an affordance is neither an objective property nor a subjective property: or it is both if you like. An affordance cuts across the dichotomy of subjective-objective and helps us to understand its inadequacy. It is equally a fact of the environment and a fact of behavior. It is both physical and psychical, yet neither. An affordance points both ways, to the environment and to the observer.”

[33] (p. 129)

The concept of affordance, as coined by Gibson, is the ecological equivalent of meaning, elaborating the idea that the meaning of a thing has a physiognomic character, as stressed already by Gestalt psychology. Koffka called this the “demand character” of things: “Each thing says what it is ... a fruit says ‘Eat me’; water says ‘Drink me’; thunder says ‘Fear me’; and woman says ‘Love me’.” [119]. Lewin, who was considered as the founder of ecological psychology, used the related term “invitation character” (*Aufforderungscharakter*) or “valence” [120] to express the meaning of things and von Uexküll [37,112,121,122,123] argued in a similar way in his early biosemiotic claims by introducing the concepts of “counter-ability” (*Gegenleistung*), “functional tone” and “functional cycle” to illustrate the importance of functional and semantic relations that biological organisms establish with their environment. In the counter-ability lies the meaning of an object for the existence of an organism. It has been elaborated more in depth in the concept of *functional tone*, which can be easily grasped through the example of a tree that has a number of different qualities or tones, dependent on the intentions that an animal or human being may confer on it. It can be a shelter for a fox, a support for the oil, a thoroughfare for the squirrel, hunting-providing grounds for the ant, egg-laying facilities for the beetle and a source of valuable raw material for the forester [37]. As such there is no one-to-one relationship between an object in the outer world and its actual meaning. Each organism, on the contrary, perceives the world through a network of functional relations, thus constituting its own phenomenal world or Umwelt, which, in turn, can be considered as the sum total of its perceptual cues among the stimuli in its environment. It is constituted of *functional cycles*, which operate by means of trigger mechanisms that select a number of objects with a special relevance to act as either perceptual or functional cue bearers. Both are related to each other in the sense that the functional qualities affect the perceptual ones—hence the concept of cycle—by transforming the object of perception by giving it a functional tone. Our relation to the world, therefore, is not merely representational, but is functional as well, which means that the number of objects, which an animal can distinguish in its own world equals the number of functions it can carry out [37] (p. 49). The objects, which an organism or animal confronts, therefore, are not neutral objects, but objects that are transformed into meaning-carriers as soon as they enter into a relationship with a subject. This is illustrated by von Uexküll’s example of an angry dog that barks at somebody on a country road. In order to drive the dog away, the person can pick up a stone, which first lies on the ground, to throw it at the dog. By doing this, he transforms its meaning and quality from a mere support for the walker’s feet (path-quality) to that of becoming a missile, thus assigning a different functional tone to it (throw-quality) [122] (p. 27).

### 5.3. Musical Affordances

Dealing with music in ecological and biosemiotic terms means that we should try to understand music not merely in terms of its acoustical qualities but in terms of what it affords to the listener [123,124,125,126,127,128]. Listeners, in fact, build up relations with the sonic world, selecting some of them to give them special meanings. As such, there is a whole history of ontogenetic development that contributes to the construction of a sonic Umwelt, which can be considered as a collection of subjective meanings that are assigned to some elements of a specific subset of the sounding environment. Music, in this view, should be described in terms of its functional signification and affordances. The question, however, is what such musical affordances are? Starting from the ecological concept of interaction with the environment, there seem at least to be three major possibilities: (i) the production of musical instruments out of sounding material; (ii) the use of playing techniques in order to produce musical sounds; and (iii) the shaping of the sound by using modulatory techniques [125].

Examples of the first are exemplified in the history of musical instrument building. About all kinds of materials have been scrutinized for what they afford to human ears from a musical point of view. This holds true for traditional instruments as well as for the many attempts at finding new sounds out of new materials. But also the development of playing techniques is related to the search for sounding materials, with a special focus on the sound-producing or sound-related actions that can be applied to them [129,130,131]. Sound-producing actions include “excitatory actions”, such as hitting, stroking, scrapping, bowing, kicking and blowing—all of them being actions that consist of transferring energy from the body to resonating objects, such as strings, plates, tubes and membranes—as well as “modulatory actions” that modify the sound, such as vibrato, opening and closing a brass mute or placing a string mute on the bridge [129]. The shaping of the sound, finally, is a further extension of sound production. Strings, e.g., can be plucked or bowed, but within the action category of bowing, there is a whole spectrum of techniques for modulation of the sound. The same holds true for singers who use their singing technique to shape the sounds that result from the air supply provided by the lungs. Singing involves not merely the production of vowels and consonants, but involves aspects of intonation and common ways of emotional expression, such as timing, articulation, dynamics, tone onsets and vibrato. It embraces, for short, the whole gamut of sentic modulation [78] with the three graded spectra of tempo modulation, amplitude modulation and selection of register.

All these examples, further, refer to the *productive aspects* of musical affordances, which take as a starting point the raw material and what it affords for musical sound production. It is possible, however, to conceive of affordances also at the *receptive level of experience* and to conceive of them in terms of perceptual, mood induction and socio-communicative qualities, which invoke aspects of sense-making, emotional experience, aesthetic experience, entrainment and judgments of value [45,127,132]. To quote Krueger: “Music, in this view, is mainly perceived as an affordance-laden structure and the musical experience is fundamentally a temporally extended, exploratory activity...” [45] (p. 2). Picking up musical affordances, in this view, is what it means to speak of music as being experienced as “something that we can do things with”, to coin his term, but it is only experienced as such by listeners with the appropriate perceptual and affective sensitivities.

It is possible, finally, to bring together productive and experiential aspects of musical affordances as exemplified in the huge body of *action and perception studies* [36]. Music, in this view, is something that induces a kind of motor resonance that prompts the listeners to experience the sounds as if they are involved in their production [133]. It is a claim, which is somewhat analogous to the central version of the motor theory of perception, which means that “motor intention” rather than “manifest motor behavior” is thought to be a largely endogenous phenomenon, which is localized in the central nervous system. It has been shown, in fact, that there is a motor aspect in perception and that the same areas in the brain are activated during imagined and executed actions (the supplementary motor area) and the same holds true for imagined action as well [134,135,136,137,138]. Perception, therefore, can be considered as simulated action, as imagining the actions that are implied in manipulating the perceived objects.

Not all perception, however, is reducible to motor components, but motor components are involved in perception and are an integral part of it [36,139]. Even if they are not manifest, they operate at virtual levels of imagery and simulation—also called *ideomotor simulation*—with motor behavior being manifest only at an ideational level of mental representation. What is argued for, therefore, is a kind of phenomenal experience, which involves the experience of movement but without the action being actual or manifest. It corresponds to the so-called internal imagery—or first person perspective—which enables the transition from overt action to internalized forms of action. The whole process calls forth a kind of motor empathy and ideomotor simulation, allowing the listener to experience the music as something that moves over time, while simultaneously experiencing this movement as a movement of the own body [133]. Musical affordances, therefore, involve an aspect of egocentricity, in describing subjective experiences in terms of bodily resonance or motor imagery that projects the listener’s bodily movements to the music.

A last interpretation of music in terms of affordances, finally, is more manifest and involves *musical entrainment* and the possibility to move in reaction to the sounding music. Music, then, is a stimulus for movement and is perceived in terms of its motor induction capacities. The movements can be specific and articulate, but they can relate also to more general levels of motor induction, as forces and energies that are inherent in musical structures.

## 6. Conclusions and Perspectives

The major aim of this contribution was to describe music as a sounding environment. Starting from an ethological approach to dealing with music, it has revolved around the ecological concept of organism/environment interaction, conceiving of the listener as an organism and the music as environment. Music, in this view, is considered as a subuniverse of the more-encompassing sonic universe with the sounding environment—as part of the sonic universe—being described not merely in objective, but also in subjective terms. As such, there is a lot of subjectivity in the delimitation of the sounding world as it appears to individual listeners, which then becomes a subjective or phenomenal world. But even if they seem to be very idiosyncratic, the phenomenal worlds are grounded also in some relatively unchanging biological processes of aural perception, which are universal across human listeners.

Music, thus, is constituted of a set of elements that may be selected and delimited at will. This can be done by acts of focal attention and conscious efforts but there is also a lot of subconscious processing and reactive behavior to the sounds. To the extent, further, that these basic constituents are considered as discrete units, it is possible to conceive of music as a Humboldt system with discrete elements that lean themselves to mental computations. This calls forth a computational approach to music with elements that refer basically to themselves, allowing a description of music in terms of internal semantics. Music, however, is also a sounding art, with aspects of meaning, which cannot be grasped merely in symbolic terms. As such, it calls forth an experiential description as well, as evidenced in the earliest ways of coping with the sounds. This is exemplified most typically by studies on fetal auditory development and auditory abilities of neonates, which are illustrative of the innate dispositions for coping with the sounds and the developmental plasticity that refers to the ontogenetic development of individual music listeners.

Listening, then, is a process of reactive behavior that is grounded in phylogenetically older levels of processing as well as higher level functioning of the brain. As such, there is a possible transition from mere sensory processing to sense-making as the outcome of a learning history, and this latter can be described in ecological and biosemiotic terms, by stressing the role of interactions with the sounds and by assigning functional meaning to these interactions. This is exemplified convincingly in the ecological and biosemiotic concepts of affordance and functional tone, which provide conceptual and operational means for the description of processes of sense-making that are the outcome of the establishment of functional relations between listener and environment.

It is possible, finally, to suggest future directions and perspectives based on the lines of thinking presented in this paper and to present a whole research program for empirical research. Some of them have started already, other ones are still waiting for implementation at a larger scale. There are at least four possible domains: (i) assessment studies, which should aim at collecting data about what listeners actually attend to while listening in a real time listening situation; (ii) methodological studies to provide the appropriate measurement tools for continuous recordings of acts of focal and distributed sense-making; (iii) setting up of a whole research program for exploring the possibilities of musical affordances, both at the productive and receptive level, somewhat analogous to the research program that was launched in the early action and perception studies [140,141,142,143,144]; and (iv) studies on the possibility of music as a sonic environment to influence social behavior. There have been some interesting experiments with music at the urban scales, e.g., by broadcasting classical music in shopping mails, parks and other public places to deter certain types of loiterers. The ethics of this, however, might be questioned.
